# The Identification of Lymphocyte-Like Cells and Lymphoid-Related Genes in Amphioxus Indicates the Twilight for the Emergency of Adaptive Immune System

**DOI:** 10.1371/journal.pone.0000206

**Published:** 2007-02-14

**Authors:** Gonghua Huang, Xiaojin Xie, Yan Han, Lifei Fan, Jie Chen, Chunyan Mou, Lei Guo, Hui Liu, Qinfen Zhang, Shangwu Chen, Meiling Dong, Jianzhong Liu, Anlong Xu

**Affiliations:** State Key Laboratory of Biocontrol, The Open Laboratory for Marine Functional Genomics of State High-Tech Development Program, College of Life Sciences, Sun Yat-sen University, Guangzhou, China; New York University School of Medicine, United States of America

## Abstract

To seek evidence of a primitive adaptive immune system (AIS) before vertebrate, we examined whether lymphocytes or lymphocyte-like cells and the related molecules participating in the lymphocyte function existed in amphioxus. Anatomical analysis by electron microscopy revealed the presence of lymphocyte-like cells in gills, and these cells underwent morphological changes in response to microbial pathogens that are reminiscent of those of mammalian lymphocytes executing immune response to microbial challenge. In addition, a systematic comparative analysis of our cDNA database of amphioxus identified a large number of genes whose vertebrate counterparts are involved in lymphocyte function. Among these genes, several genes were found to be expressed in the vicinity of the lymphocyte-like cells by *in situ* hybridization and up-regulated after exposure to microbial pathogens. Our findings in the amphioxus indicate the twilight for the emergency of AIS before the invertebrate-vertebrate transition during evolution.

## Introduction

The emergence of adaptive immunity represents a major step in the host-pathogen arm race that has led to the current highly elaborate immune response system in vertebrates. The identification of lymphocyte-like cells and molecules participating in the immune response and recognition processes in lamprey [1], the oldest living jawed vertebrate, suggests the emergency of adaptive immune system (AIS) before or shortly after the dawn of vertebrate evolution approximately 500 mya ago. However, it remains unclear exactly when AIS first appeared during evolution.

Recently, a few homologs of vertebrate genes involved in AIS have been identified in Amphioxus [2]–[4], a cephalochordate that was considered as a living invertebrate most closely related to vertebrate [5] and has recently been thought as the ancestor of all deuterostomes [6], [7], suggesting that the evolution of these basic components of AIS, or perhaps AIS may predate the invertebrate-vertebrate transition. Here, we report morphological and functional evidence for the presence of lymphocyte-like cells in Chinese amphioxus. In addition, we provided a comprehensive list of amphioxus genes whose mammalian homologs are involved in certain aspects of lymphocyte-based immune response based on a systematic analysis on our cDNA database [8]. The findings of lymphocyte-like cells and their related genes in amphioxus represent initial appearance of AIS before the invertebrate-vertebrate transition during evolution.

## Results

### Identification of lymphocyte-like cells in amphioxus

**Figure 1:**
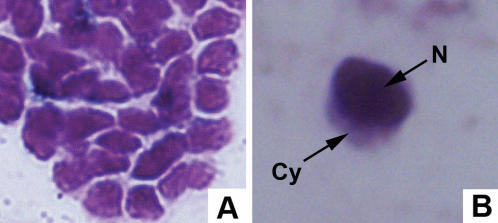
Light-microscopic views of the lymphocyte-like cells in the amphioxus. (A) Many lymphocyte-like cells in the gills. Magnification 400. (B) The cells of the amphioxus gills contained large, darkly stained nuclei and the thin rim of cytoplasm. Magnification 1000, Wright stained.

**Figure 2:**
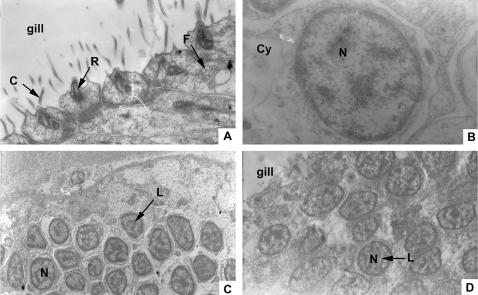
Transmission electron-microscopic views of the lymphocyte-like cells in follicle-associated epithelium of amphioxus gill. (A) Follicle-associated epithelium cells in the gill contained follicle (F) rootlet (R), and cilia (C). Magnification 29000. (B) The lymphocyte-like cells contained large nuclei (N) with heterochromatin forming a peripheral rim adjacent to the nuclear envelope. Magnification 48000. (C) Under the FAE of normal amphioxus gill, lots of lymphocyte-like cells (L) were seen. The cells contained large nuclei (N) with heterochromatin forming a peripheral rim adjacent to the nuclear envelope. Magnification 5800. (D) At the same magnification, after the microbial challenge, the lymphocyte-like cells were bigger than those of normal cells.

It has been previously shown that lymphocyte-like cells are present in the intestine-associated typhlosole of lamprey [9]. To examine whether amphioxus has lymphocyte-like cells, we dissected the gills of amphioxus under light microscopy and observed that a cluster of cells contained large, darkly stained nuclei and a thin rim of cytoplasm, which were highly reminiscent of mammalian lymphocytes (Figure 1). We further used the electron microscope to see the structure of the gills and observed the well-organized mucosa-associated structures surrounded by follicle epithelium in the gill. The follicle epithelium cells possessed cilia and rootlet structure (Figure 2A), which was described by Ratcliffe and his colleagues [10]. The cluster of the cells observed was surrounded by the follicle epithelium with dense and small round cells with certain characteristics of lymphocytes. For example, each of these cells contained a large nucleus (N) with heterochromatin forming a peripheral rim adjacent to the nuclear envelope surrounded by a thin layer of cytoplasm (Figure 2B). When adult amphioxus was challenged by pathogenic bacteria, the size of the lymphocyte-like cells increased remarkably compared to those in the unchallenged control (Figure 2C, 2D), indicating the morphological changes of the lymphocyte-like cells in response to the challenge of pathogen.

### The identification of lymphoid-related homologs in amphioxus by comparative genome analysis

**Figure 3:**
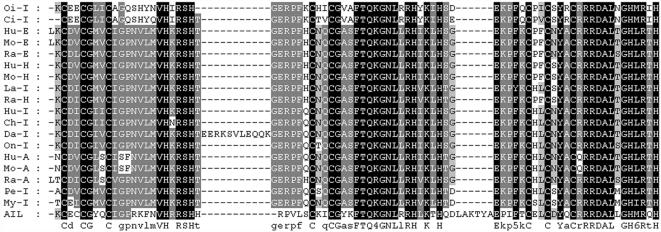
Amino acid alignment of the first to third zinc finger domains in Ikaros family members. GenBank accession codes of the corresponding nucleotide sequences are: U40462, *Homo sapiens* (*Hu*-I); Y11833, *Gallus gallus* (*Ch*-I); U92198, *Onchorynchus mykiss* (*On*-I); AF092175, *Danio rerio* (*Da*-I); AF192380, *Petromyzon marinus* (*Pe*-I); AF424735, *Lampetra fluviatilis* (*La*-I); AY237104, *Myxine glutinosa* (*My*-I); AY237106, *Oikopleura dioica* (*Oi*-I); *Ciin* (*Ci*-I). Ikaros have the model identity numbers 144428 (18 ); AF130863, *human Helios* (*Hu*-H); AF044257, *mouse Helios* (*Mo*-H); AF163847, *Raja eglanteria Helios* (*Ra*-H); AF129512, *human Aiolos* (*Hu*-A); AF001293, *mouse Aiolos* (*Mo*-A); AF163850, *Raja eglanteria Aiolos* (*Ra*-A); AF230809, *human Eos* (*Hu*-E); AB017615, *mouse Eos* (*Mo*-E); AF163849, *Raja eglanteria Eos* (*Ra*-E); AIL: Ikaros-like gene from amphioxus.

**Figure 4:**
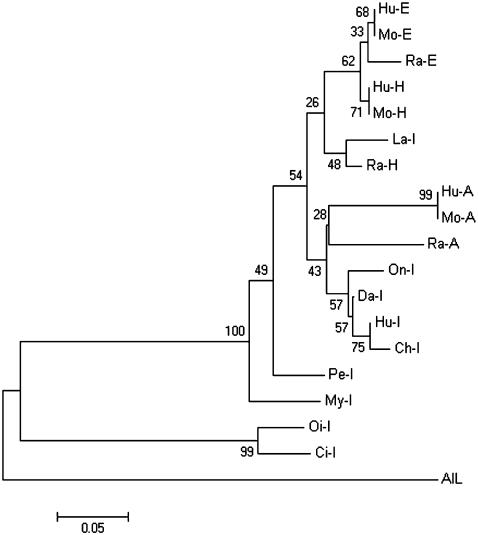
Neighbor-joining tree of ikaros proteins. The tree is based on the amino acid alignment in Figure. 3. Numbers on the branches indicate bootstrap values. The tree is rooted at midpoint.

**Figure 5:**
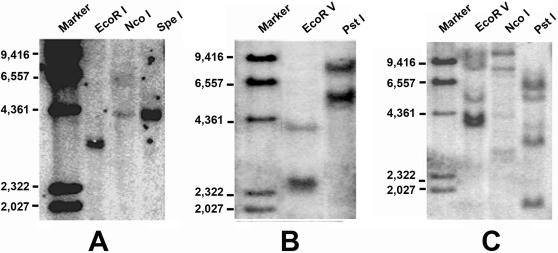
Southern blot analysis. Genomic DNA from *Branchiostoma belcheri tsingtauense* was digested with restriction enzymes as indicated. The blot was hybridized with a probe at high stringency with the full-length of Ikaros-like, Bam32 and TAPP1 from amphioxus.

**Figure 6:**
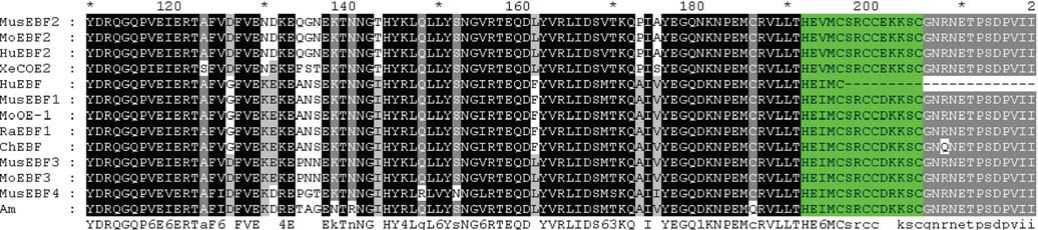
Alignments of DNA binding domain. The zinc finger motif was colored with green. The consensus sequence was under the alignments. Accession number for the COE family members are as following: NP_034225, MusEBF2; NP_073150, HuEBF2; AAH41178, HuEBF; NP_031923, MusEBF1; Q07802, MoOE-1; NP_446272, RaEBF1; NP_990083, ChEBF; NP_034226, MusEBF3; NP_694538, MusEBF4; O08791, MoEBF3.

**Figure 7:**
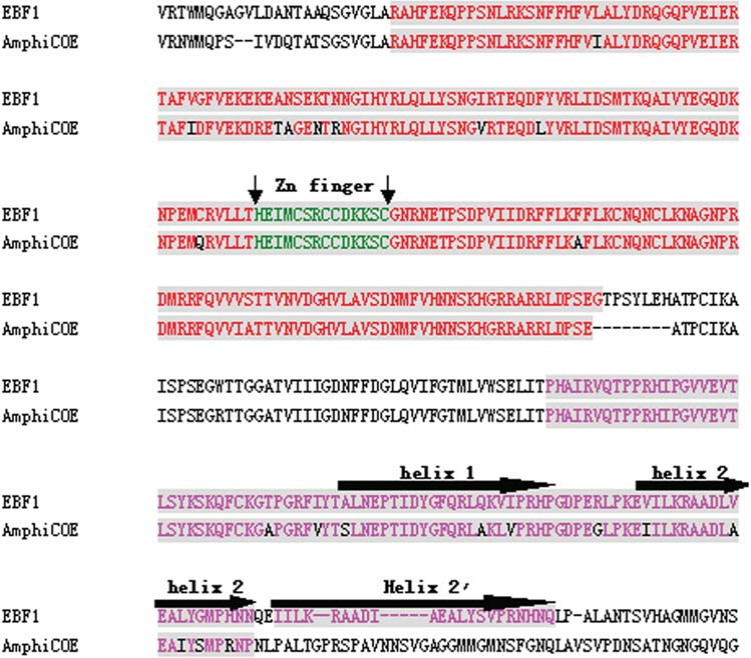
Alignment of Mus EBF1 and amphioxus EBF protein sequence. The conserved DNA binding domain and IPT/TIG domain were colored with red and orange separately. The atypical zinc finger motif obligatory for DNA binding was marked with black arrows. The horizontal dashed arrow indicated the helix 1 and helix 2 of HLH motif. The additional duplicated helix 2 in vertebrate COE proteins was also indicated. The accession number of Mus EBF-1 is NM_007897.

**Figure 8:**
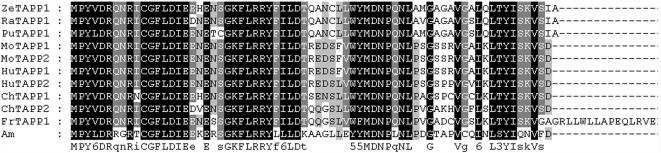
Amino acid sequence alignment TAPP1 and TAPP2. Hu = human, Mo =  mouse, Ch = chicken, Am = amphioxus, Fr = frog, Ze = zebrafish, Ra = rat, Pu = Pufferfish. NCBI accession numbers: Q9HB21, human TAPP1; AF286164, human TAPP2; Q8BUL6, mouse TAPP1; Q9ERS5, mouse TAPP2; XP_421799, chicken TAPP1; NP_990029, chicken TAPP2; AAH76776, frog TAPP1; AAH44452, zebrafish TAPP1; XP_341943, rat TAPP1; CAG10059, Pufferfish TAPP1.

**Figure 9:**
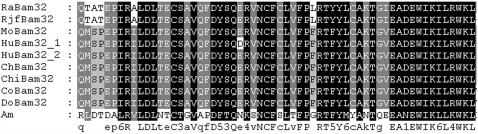
Amino acid sequences alignment of Bam32 from various speices. Mo = Mouse, Hu = Human, Ch = chicken, Chi = chimpanzee, Co = cow, Do = dog, Ra = Norway rat, Rjf = red jungle fowl, Am = amphioxus. NCBI accession numbers: Q9QXT1, MoBam32; NP_055210, HuBam32.1; Q9UN19, HuBam32.2; CAG32077, ChBam32; XP_517361, ChiBam32; XP_612467, CoBam32; XP_535669, DoBam32; XP_342349, RaBam32; XP_423213, RjfBam32.

The presence of lymphocyte-like cells in amphioxus prompted us to examine to what extend this primitive organism has acquired the various components associated with lymphocytes. To address this issue, a systematic comparative analysis of amphioxus cDNA database was carried out to identify the homologs or orthoglogs which in the more advanced organisms are involved in lymphocyte-based immunity. This study resulted in the identification of an extensive number of candidates (Table 1). Among those genes, the Ikaros-like gene, early B-cell factor (EBF/COE), B lymphocyte adaptor molecule of 32 kDa (Bam32) and tandem PH domain-containing protein (TAPP1) were noticeably identified. The Ikaros-like gene in amphioxus contains 1725 bp and encodes 276 amino acids. The homologous analyses showed that the initial 3 C2H2 zinc domains with the DNA-binding function had 50% homology to the typical Ikaros [11] (Figure 3). On the phylogenetic tree of available ikaros and related sequences, the amphioxus sequence assumes the position in the ikaros clade expected on the basis of its taxonomical origin (Figure 4). Southern blot results indicated that there was only one copy of Ikaros-like gene in the amphioxus genome (Figure 5A). The EBF identified in amphioxus contained 5400 bp and encoded 634 amino acids. Homologous analysis indicated that the DNA-binding domain (DBD) of amphioxus EBF had a high homology with other EBF proteins, especially in the zinc-binding domain (Figure 6). Following the DBD domain, a conserved IPT/TIG domain, which was located in 283–369 amino acids, could be found and had 91% homology with the EBF3 in human. Following the IPT/TIG domain, a conserved helix/loop/helix (HLH) domain could be found (Figure 7). Bam32 and TAPP1 in amphioxus encoded 318 amino acids and 250 amino acids, respectively. Alignment result showed that the first N-terminal 30 amino acids in our sequence were remarkably well conserved to those of vertebrate TAPPs (Figure 8). Homologous analysis indicated that amphioxus Bam32 was highly homologous to the Bam32 of other selected species (Figure 9). Southern blot hybridization indicated that Bam32 was present in two-copies in the Chinese amphioxus genome while TAPP1 more than four-copies (Figure 5B, 5C).

**Table 1:** Comparative analyses of some lymphoid-related genes in our database in different organisms

Similarity to	Nematode	Fruit Fly	Ascidian	Amphioxus	Zebrafish/Fugu	Mouse	Human
Ikaros-like	+	+	+	+	+	+	+
BCAP	−	+	−	+	+	+	+
Bam32	−	−	−	+	+	+	+
TAPP1	−	−	−	+	+	+	+
ETS	+	+	+	+	+	+	+
EBF	+	+	+	+	+	+	+
CXC-R3	−	−	−	+	+	+	+
CD9/CD81	+	+	+	+	+	+	+
CAST	−	−	+	+	+	+	+
CD75	−	+	+	+	+	+	+
CD45	−	+	−	+	+	+	+

### The expression of the lymphoid-related genes in amphioxus

**Figure 10:**
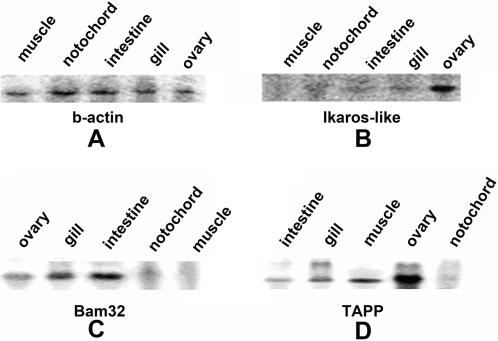
Nouthern blot analysis. Total RNA from muscle, notochord, intestine, gill and ovary of amphioxus were 20ug in each lane. (A) amphioxus b-actin. (B) amphioxus Ikaros-like. (C) amphioxus Bam32. (D): amphioxus TAPP1.

**Figure 11:**
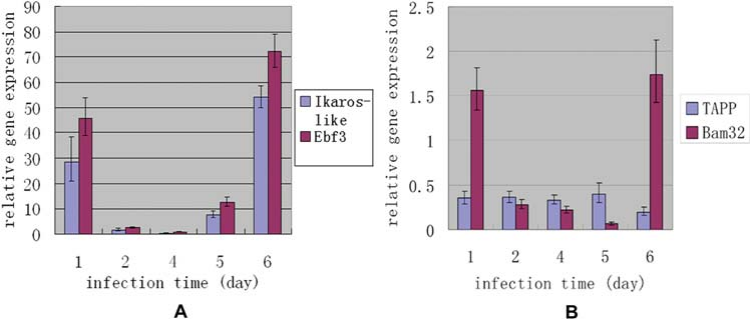
Analysis of gene temporal expression pattern by Real-time PCR analysis. Amphioxus mRNA extracted at 1 d, 2 d, 4 d, 5 d and 6 d after the *V.p* injections were used for Real-time PCR analysis. β-actin gene of amphioxus was used as endogenous control.

The identification of both lymphocyte-like cells and lymphocyte-related genes prompted us to further characterize the expressions of those four genes. We first used Northern blot to examine the main organs in which these genes expressed, and found expression of Ikaros-like in ovary and gills, especially in the ovary (Figure 10B), which was consistent with those three Ikaros-like gene family found in lamprey [12], but different from those in the higher vertebrate (mainly in the lymphoid tissues). We also detected Bam32 in ovary, gill and intestine, with the highest levels in gill and intestine (Figure 10C). In addition, we also found that the amphioxus TAPP1 transcript was expressed in all tissues tested except notochord, with the strongest expression in ovary (Figure 10D). We further used Real-time PCR to study the expression of these genes with or without challenge by microbial pathogens, demonstrating that 4 days after challenge the expressions were gradually down-regulated, but up-regulated again after the fifth day (Figure 11A, 11B).

**Figure 12:**
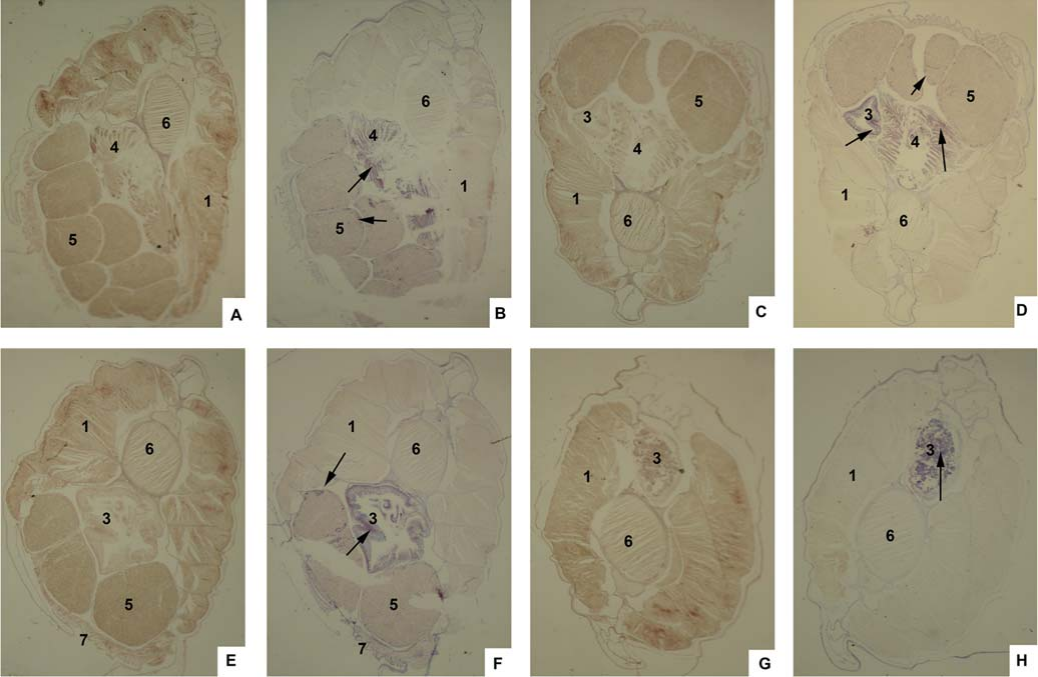
Analysis of gene spartial expression pattern by *in situ* analysis. Amphioxus was fixed with 4% paraformaldehyde. Horizontal sections of these samples were hybridized with the sense probes of Ikaros-like (A), EBF (C), Bam32 (E), and TAPP1 (G) as the control. (B) The Ikaros-like genes expressed in the ovary and gills. (D) The EBF gene expressed at the gills, ovary and intestine. (F) Expression of Bam32 was in ovary, metapleural fold and intestine. (H) Expression of TAPP1 was observed in intestine. In all sections, tissues and organs are indicated as flowed: 1, muscle; 2, hepatic cecum; 3, intestines; 4, gill; 5, sexual-gland; 6, notochord. 7, metapleural fold. Positive signals are shown by arrow.

Then, we examine whether these genes were expressed in the place as it would be expected if they were indeed involved in response to the microbial challenge that was mediated by these cells. Results of *in situ* analysis showed that indeed all the mRNA of all these genes could be detected in the first immune defense line where the lymphocyte-like cells played their roles. Specifically, Ikaros-like mRNA was detected in gills and ovary (Figure 12B); EBF mRNA was detected in gills and intestine (Figure 12D); Bam32 mRNA was detected in gill, ovary and metapleural fold. (Figure 12F); TAPP1 positive cells were detected with strong signals in intestine (Figure 12H).

### Functional comparison of Amphioxus Bam32 and TAPP1 with their vertebrate homologs

**Figure 13:**
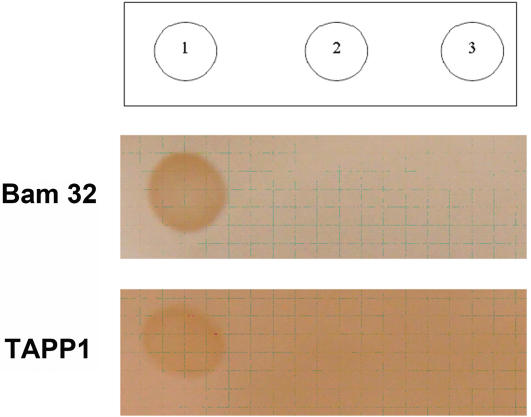
Phosphoinositide binding properties of the amphioxus Bam32 and TAPP1. The ability of the following GST fusion proteins of amphioxus Bam32 and TAPP1 was analysed using a protein-lipid overlay. The indicated phosphoinositides (20 pmol) were spotted on to nitrocellulose membranes, which were then incubated with the purified GST-fusion proteins. The membrane was washed, and the GST-fusion proteins bound to the membrane by virtue of their interaction with lipid were detected using a GST antibody. A representative experiment of three is shown. The phosphoinositides are identified by the positions of encircled numbers at the top of the figure. 1, PtdIns (3, 4) P2; 2, PtdIns (3, 4, 5) P3; 3, PtdIns.

The common ancestors of vertebrates and amphioxus diverged around 600 million years ago. To determine whether Bam32 and TAPP1 from amphioxus perform the same functions as their counterparts from vertebrates, we used a protein-lipid overlay assay to study the interaction between these proteins and phospholipids. Our results demonstrated that the Bam32 and TAPP1 GST fusion proteins all interacted with PI (3, 4) P2, but not with PI (3, 4, 5) P3 and PtdIns (Figure 13), suggesting that the functions of these two primitive genes were consistent with those in vertebrate [13].

## Discussion

The evolutionary origin of acquired immune mechanism has been one of focal interests in immunology for a long period. Last years, homology searching for the vertebrate AIS-specific molecules in amphioxus had made some important progress [8]. However, as the vital cells for AIS, lymphocyte in this animal was still not identified although it has been identified in all species of jawed vertebrates [14] and jawless vertebrate recently [9].

The immune system is functionally compartmentalized into primary lymphoid organs and secondary lymphoid tissues in vertebrates. The primary lymphoid tissues are the place where lymphocyte precursors develop into immunocompetent naive lymphocytes. Secondary lymphoid tissues are the spleen, lymph nodes, and organized lymphoid tissues associated with mucosal surfaces, including the tonsils, bronchial-associated lymphoid tissues, gut-associated lymphoid tissues, Peyer's patches, and other less-prominent organized clusters of lymphoid cells associated with the gastrointestinal, genitourinary, and respiratory tracts. These lymphoid tissues are located at strategic sites where foreign antigens entering the body from either the skin or a mucosal surface can be trapped and concentrated [15]. Thus far, no distinct lymphoid tissues have been found in agnathan and protochordate by histological studies but some lymphocyte-like cells were isolated from the intestine and the associated typhlosole of lamprey [9]. In our observation of amphioxus under the electron microscopy, though the primary lymphoid organs were not found, the organized mucosa-associated lymphoid tissues (MALTs) were found in the amphioxus gills with the hallmark of MALTs as the presence of lymphoid follicles. Many lymphocyte-like cells were seen at the MALTs, and all cells fit the description of lymphocytes by a number of criteria. First, the amphioxus lymphocyte-like cells are morphologically similar to mammalian lymphocytes under light or electron-microscopy. They also possess physical characteristics of lymphocytes, such as large nuclei with heterochromatin forming a peripheral rim adjacent to the nuclear envelope. The nucleus is surrounded by a thin layer of cytoplasm. Second, the amphioxus lymphocyte-like cells are abundant in tissues such as gut (data not shown) and gills, the first defense line against the foreign pathogens in amphioxus. Third, when challenged with microbial, the sizes of those cells were enlarged. Fourth, the gene products of mammalian homologs for these four identified genes are involved in lymphocyte development [11], B-cell lymphopoiesis [16], [17] and as the adaptor proteins in lymphocyte activation [13], [18] respectively. Moreover, these genes were up-regulated after the microbial challenge (TAPP1 was slightly down-regulated) and were expressed mostly in gill, ovary, hepatic cecum and intestine, which is co-localized with the tissue location of lymphocyte at the right time and at the right place in amphioxus. The interaction of amphioxus Bam32 and TAPP1 with PI (3,4,5) P3 and PI (3,4) P2 with preference with PI (3,4) P2 as a binding partner *in vitro* was found as observed previously in vertebrate lymphocytes [13], further substantiating the finding of functional lymphocytes in amphioxus along with the expression profiles of the lymphocytes-restricted molecules.

In summary, though our results do not provide the directive evidence for the emergency of AIS in the amphioxus, the identification of lymphocyte-like cells and lymphocyte-related genes in this animal indicates the twilight for the emergency of fully functional AIS.

## Materials and Methods

### Animals and cells

Mature adults of Chinese amphioxus, *B. belcheri tsingtaunese*, were obtained from Kioachow Bay near Qingdao, China, and cultured in our laboratory (at 25°C) filled with air-pumped circulating infiltrated seawater. The gills of the amphioxus were dissected under the light microscopy. Cells harvested by maceration of these tissues between two glass slides were suspended in PBS. The cells were filtered and then centrifuged at 1000 g for 10 min, then stained with Wright's staining, washed, and photographed.

### Structural analysis of amphioxus gill using electron microscopy

Total 5 µl (10^8^ cells/ µl) live *V.p* PBS suspensions were microinjected into the gut of amphioxus. Animals injected with PBS only were used as the controls. Amphioxus gills were dissected under the microscopy after challenged 5 d and was fixed by 3% glutaraldehyde, washed with PBS then cut into small pieces, dehydrated in graded series of ethanol and finally dehydrated in acetone and fixed in osmium tetroxide and embedded in spur. Ultrathin sectioning was used for uranyl acetate and lead citrate staining for microscopic analysis.

### Full length ESTs obtained using 3′ walking sequencing

The partial cDNA sequences of Ikaros-like, EBF, Bam32 and TAPP were obtained from different tissues cDNA libraries which had been constructed in our lab [8], [19]. The full lengths of those genes were obtained using 3′ walking sequencing.

### Northern blot hybridization

Total RNA from various tissues of amphioxus was extracted and denatured by glyoxal at 50°C for 1 h, and separated electrophoretically on a 1% agarose gel. The gel was blotted onto a Hybond-N^+^ nylon membrane (Amersham Biosciences) using 20×SSC. The membranes crosslinked with ultraviolet light and baked at 90°C for 2.5 h. Hybridization was at high stringency in Modified Church and Gilbert buffer overnight at 65°C. The membrane were washed in 1×SSC, 0.1% SDS at RT before exposed to X-ray film (Eastman Kodak) for 30 h at −80°C.

### Southern blot hybridization

Genomic DNA (16 µg/enzyme-reaction) was digested with *Eco*R V, *Spe* I and *Nco* I, respectively. Digested genomic DNA was fractionated by 0.8% agarose gel electrophoresis and transferred to Hybond N^+^ nylon membranes (Amersham Biosciences) overnight by alkali blotting. After hybridization at 42°C overnight, the membrane was washed twice in 2× SSC-0.1% SDS at room temperature and two times in 0.5× SSC-0.1% SDS at 68°C. Blots were autoradiographied for empirically optimized exposure times.

### Expression analysis by quantitative PCR

In order to further study the functions of those identified genes in the cDNA library, we re-infected the amphioxus with the *V.p*. Total RNA was prepared using TRIZOL reagent (Invitrogen) from the independently infected amphioxus at 1 d, 2 d, 4 d, 5 d and 6 d after the injection. After digested with DNase I (RNase free, Takara) to eliminate the genome contamination, the cDNA was synthesized with the Superscript^III^ reverse transcriptase (Invitrogen) using the oligo d(T) primer. Real-time PCR was performed with the ABI PRISIM 7900 sequence detection system. SYBR green Real-time PCR mix (Toyobo) was used for PCR reaction, with a primer concentration of 200 nM. Reaction conditions consisted of 95°C for 1.5 min, followed by 40 cycles of 95°C for 15 s, 55°C for 15 s, 72°C for 1 min. Reaction of each sample was performed in triplicate. Amphioxus β-actin was used as control to normalize the starting quantity of RNA. Standard curves were constructed for target genes and β-actin with two-fold serial dilutions of cDNA. The threshold cycles and fold inductions were calculated by the ABI PRISIM 7900HD SDS software. After Real-time PCR, the products were analyzed on 1.5% agarose gel.

### *In situ* hybridization

Adults of the Chinese amphioxus, *B. belcheri tsingtauense*, were collected and kept in filtered seawater for 2 days. The animals were killed and cut at 1-cm intervals. The tissue blocks obtained were fixed in 4% paraformaldehyde in PBS and embedded in paraffin. Tissue blocks were cut transversally and mounted on glass slides coated with poly-lysine. The digoxigenin-labeled probes were prepared by using the plasmids that contain the sequences of the interesting gene with the SP6 promoter sequence at the 3′ end of the sequences as template and the antisense probes were synthesized with the SP6 RNA polymerase according to the protocol of the digoxigenin DIG RNA labeling kit (Roche). *In situ* hybridization was performed according to Li et al. [20] with a litter modification. The sense probes were used as the control.

### GST fusion proteins and protein-lipid overlay

cDNAs encoding Bam32 and TAPP1 were cloned into pGEX-4T-2 (Amersham) to obtain the production of Bam32 and TAPP1 GST-fusion proteins for the phospholipid-binding analysis using a protein-lipid overlay. Briefly, small scale cultures were induced in exponentially growing bacteria (A600 = 0.6–0.8) with 0.1 mM isopropyl -D-thiogalactoside for 20 h at 18°C. Fusion proteins were then purified from the lysates using glutathione-Sepharose spin columns (Amersham). Lipid solution (2 µl) containing 10pmol of phospholipids, PtdIns (3, 4)P_2_, PtdIns (3,4,5)P_3_ or PtdIns (Sigma), dissolved in a mixture of chloroform/methanol/water(1∶2∶0.8) was spotted onto Hybond-C extra membrane and allowed to dry at room temperature for 2 h. The membrane was blocked in 3% (w/v) fatty acid-free BSA in PBST [137 mM NaCl, 2.7 mM KCl, 10 mM Na_2_HPO_4_, 2 mM KH_2_PO_4_, 0.1%(v/v) Tween-20] for 2h and then incubated overnight at 4°C in the same solution containing 50 µg/ml Bam32-GST or TAPP1-GST fusion protein and washed five times over 40 min in PBST, and then incubated for 1h with a 1/2500 dilution of anti-GST monoclonal antibody (Novagen). After washed, the membrane was incubated for 1 h with a 1/200 dilution of anti-mouse-horseradish peroxidase conjugate. Finally, the GST-fusion protein bound to the membrane, by virtue of its interaction with phospholipid, was detected.

### Bioinformatics analysis

Full length cDNA sequences of genes were searched using the BLASTX algorithm against NR Database from the National Center for Biotechnology Information (NCBI). The homologous sequences were selected from the BLASTX search results as the input for phylogenetic analysis. The input data for phylogenetic methods were based on the most consistent alignment obtained with the CLUSTALW program and reduced by GBLOCKS. The phylogenetic analysis was conducted using the Mega3.0 software.
